# Identification of Explanatory Variables in Possession of the Ball in High-Performance Women’s Football

**DOI:** 10.3390/ijerph18115922

**Published:** 2021-05-31

**Authors:** Rubén Maneiro, José Luís Losada, Claudio A. Casal, Antonio Ardá

**Affiliations:** 1 Department of Science of Physical Activity and Sport, Pontifical University of Salamanca, 37007 Salamanca, Spain; 2 Department of Social Psychology and Quantitative Psychology, University of Barcelona, 08001 Barcelona, Spain; jlosada@ub.edu; 3 Department of Science of Physical Activity and Sport, Catholic University of Valencia “San Vicente Mártir”, 46900 Valencia, Spain; ca.casal@ucv.es; 4 Department of Physical and Sport Education, University of A Coruña, 15172 A Coruña, Spain; ardasd@udc.es

**Keywords:** performance analysis, observational methodology, women’s football, female soccer, decision trees, logistic regression

## Abstract

Women’s football is a phenomenon that is experiencing an unstoppable increase in recent years. The objective of this study was to analyze how ball possessions are performed in high-performance women’s football. For this, the 52 matches played by all the national teams participating in the Women’s World Cup 2015 were analyzed. A total of 3740 ball possessions were examined. Using the “move outcome” variable as a reference criterion, a statistically significant relationship was found between possessions that ended in success and possessions that have failed. Specifically, the successful possessions were those that were made in the offensive zone, with a clear intention to progress towards the rival goal, with a low number of passes, and made in the second half of the matches. The results of the logistic regression showed that the proposed model is statistically significant, with an acceptable explanatory capacity. Finally, the results of the decision tree evidence the success of those possessions aiming at a quick completion, with very few passes and the offensive zone as a priority area for the possession.

## 1. Introduction

In professional male football, a goal is the most important performance indicator. Getting more goals than the opponent is synonymous with victory during the matches. The success or failure of this sport comes from the victories achieved during the competition. On the other hand, and unlike other sports such as handball or basketball, football is a low or very low scoring sport [1,2]. In addition, studies are finding that, in recent years, fewer goals are scored, and that these are associated with very specific moments or circumstances of the match [3,4].

This circumstance has led to a debate in the scientific literature. Researchers began to inquire about what can be the mechanisms that potentially increase goal achievement during the matches [5,6,7], to be able to improve sports performance in the applied field. Without a doubt, finding a formula that can predict the highest level of performance in football has emerged as the maximum aspiration of any researcher in this field. However, in most of the published works, there is not yet a scientific consensus backed by data and research that allows to issue a formula on how to reach the goal; that is, the goal in football is a highly random objective, with a high associated entropy, and where there are many inputs that must be controlled to be able to issue results accurately. Scientific research in this sport is still a long way from predicting a goal, and doing so conclusively.

Therefore, researchers have begun to establish small links to success as an alternative to a goal. These links are performance indicators, and some of the most important ones were proposed by Hughes and Bartlett, [8], although previous studies had begun to mention them [8,9]. In the words of the authors themselves, performance indicators can be defined as a selection, or combination, of action variables that aims to explain some or all aspects of a performance. Clearly, to be useful, performance indicators should relate to successful outcomes or performances. As an alternative to a goal, they mention that some of the performance indicators associated with football are the number of shots to goal, the pace of attack, the length of passes, dribbling or ball possession.

One of the most important and most studied performance indicators in the scientific literature in male soccer has been ball possession [10,11,12,13,14,15,16,17,18,19,20,21,22,23,24,25,26,27,28,29,30,31,32]. Analyzing all these studies and their relationship with possession shows that there is not yet a scientific consensus that allows to establish robust recommendations for men’s football teams. We still do not have conclusive data on whether possession is necessary to achieve victory, or something not related to success. Currently, ball possession is considered a complex phenomenon [26], and for this reason there is still more literature needed in this field. There are many variables that have been related to this performance indicator, from the many championships that have been studied. Thus, variables such as match status, playing at home or as a visitor, the quality of the opposing team, the ball possession zone, which are the players that are more recommended to make possession, the type of championship considered and even the time period analyzed, have been variables that have modulated this performance indicator.

In this way, some studies that have related the possession of the ball with the match status have shown that the teams modify their possessions depending on whether they are winning, drawing or losing on the scoreboard. On the one hand, the works of Jones et al. [27], Lago and Martín [14], Lago-Peñas [30], Lago-Peñas and Dellal [13] and Barreira, Garganta and Anguera [16] show that the teams that are losing on the scoreboard have longer possession periods. In contrast, the studies of Bloomfield et al. [11], Taylor et al. [31], Casal et al. [23] and Maneiro et al. [25] disagree with these results, affirming that the longest possession time is carried out by the teams that are winning. Some of the explanations presented by the authors to explain these differences could be found in the playstyle adopted according to the match status, or the type of competition analyzed.

On the other hand, playing at home or as a visitor (match location) has also been evidenced as a situational variable that modulates the possession of the ball in men’s football. Thus, it has been shown that the teams that play at home have a higher percentage of ball possession than the visitors [14,30,31,32]. Some possible explanations for this behavior may be due to the responsibility that the teams adopt when they compete in their field, which invites them to maintain possession of the ball and the initiative in the game.

The ball possession zone has been another performance indicator considered in the scientific literature. Again, we find debate in the studies considered. On the one hand, it has been shown that the teams that are winning on the scoreboard have greater ball possession in defensive areas of the field [16,30]. Other works affirm that the possession of the ball in offensive zones is a good predictor of success for the teams [21], and that successful teams have possessions in the middle offensive zone, while the unsuccessful ones have possessions in the medium defensive zone. In addition, recent works have found a statistically significant relationship between the success of the teams, and the variability of the playing space in ball possession [33].

On the other hand, studies that have analyzed the possession of the ball based on the winning or losing teams at the end of the game, have confirmed that successful teams have more possession of the ball in general than unsuccessful ones [11,21,25,27].

The quality of the rival team is another situational variable that has been related to ball possession in high-performance football. Specifically, the ball possession time is longer when teams face low-level or lesser-quality rivals [11,14,27,30].

Finally, a recent work has also analyzed the individual possession that players make within the overall context of the team. Thus, recent works, such as Link and Hoernig [34], state that the longest possession time is done by the goalkeepers and the central defenders; the study also finds a high ball possession rate for midfielders, a result that is supported by other studies of a similar nature [35,36,37].

Regarding the analysis of tactical aspects in women’s football, it should be mentioned that there are few studies available. According to Kirkendall and Urbaniak [38], only 25% of football-related publications focus on women’s football. Some of the studies published on tactical aspects [39,40,41] focus on the differences between both types of football. Specifically, it was shown that the duration of the attack, as well such as the starting area of this, were significant criteria when evaluating the probability of obtaining offensive success by the teams analyzed. On the other hand, the study by Kubayi and Larkin [42] showed that the winning teams presented better data in the variables related to technical performance, such as the number of passes per game, data that is supported by the work of Casal et al. [43].

In view of these data, it is possible to verify that the possession of the ball as a performance indicator is being widely studied in men’s football, and that due to its complex and multifactorial nature, the debate in the scientific literature is a constant one. On the other hand, ball possession in women’s football has not yet been analyzed. Previous studies are very limited, and their results unclear. In addition, studies of a multivariate nature in women’s football are very scarce. The objective of this study was to analyze and acknowledge how ball possessions are performed in high-performance women’s football. The ball possessions that were made in the FIFA Women’s World Cup 2015 were analyzed, and three types of analyses were performed, corresponding to three specific objectives: first, a univariate analysis with the categorical and continuous variables selected; at the bivariate level, different χ^2^ tests were made to find out the variables that had a significant relationship with the possession of the ball; and finally, through the realization of a logistic regression and the implementation of the decision tree technique, different predictive models were established.

## 2. Materials and Methods

### 2.1. Design

Among the possible designs that an observational methodology can present, a nomothetic, intersessional follow-up and multidimensional design was applied [44,45]. The systematic observation carried out was non-participant and active, using an observational sampling “all occurrence”.

### 2.2. Participants

In order to control the situational variables that could be affecting ball possession between successful and unsuccessful teams, all matches corresponding to the FIFA Women’s World Cup 2015 were analyzed. In this study, the unit of analysis is ball possession in high-level football. Encounters were recorded from public images broadcasted on television, and through a post-event record, thus ensuring respect for behavior spontaneity, as well as the registration in its natural environment. According to the Belmont Report [46], the use of public images for research purposes does not require consent.

The observation sample was a convenience sample [47]. In total, 3740 events were collected, corresponding to the observation of the 52 games that make up the world championship; specifically, the group stages, round of 16, quarterfinals, semifinals and final. These matches are disputed in the direct elimination mode, which causes both teams to need offensive attack procedures to achieve a positive result.

### 2.3. Instruments

The observation instrument proposed by Maneiro et al. [25] has been used and can be found in Table 1.

### 2.4. Procedure

There were four observers selected for data collection, three of them being PhDs in Physical Activity and Sports Sciences and a national football coach, with more than five years of experience in the use and application of observational methodology. In addition, two of the authors are methodologists and expert in observational methodology, with years of experience and relevant publications in the field [48].

Prior to the coding process, the observers were trained during eight training sessions [49], applying the consensual agreement criterion among observers and they were provided with a specifically designed observation protocol. Data quality control was carried out using the IBM SPSS Statistics 25 program by means of an interobserver concordance analysis by Cohen’s Kappa coefficient [50] for each of the criteria, the overall value being very good (0.83) according to the scales of Fleiss, Levin and Paik [51].

### 2.5. Statistical Analysis

The statistical calculation program used was R, using the libraries “compareGroups” [52], “rpart” [53] and “partykit” [54]. A recategorization of the variable “move outcome” was performed, which consisted of transforming its four categories—goal, shot, sending to the area and no success—into two categories: the category NO SUCCESS (NE), which already existed, and the SUCCESS (E) category, which included the other three, namely, goal, shot and sending to the area. A descriptive analysis of all the variables was carried out at the same time as a bivariate study between the explained variable “move outcome” and the rest of the variables that make up the observation instrument. Predictor variables that showed a significant relationship with the explained variable and its effect size were studied. This information was subsequently used to configure a logistic regression model.

**Figure 1:**
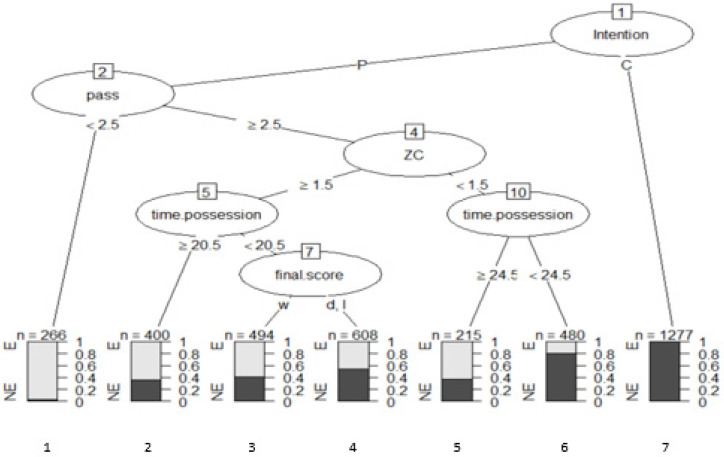
Graphic representation of the model.

As a final analysis, a multivariable technique based on decision trees was incorporated to know what the probabilities of NO SUCCESS and SUCCESS were in the different combinations (Figure 1). It is a nonparametric approach; that is, without supposed distributions. It has an easy control of lost values and strongly asymmetric data without the need to resort to data transformation. It is a robust analysis of outliers and, in addition, allows the analysis of sequential decisions based on the use of associated probabilities. For this, the Chi-square automatic interaction detector (CHAID) was used as a growth method, which consists of a statistical and multidirectional tree algorithm that scans data quickly and efficiently, and creates segments and profiles compared to the desired result. In each step, CHAID chooses the predictor variable that presents the strongest interaction with the explained variable. The categories of each predictor merge if they are not significantly different from the predictive variable.

## 3. Results

In the descriptive analysis, for the categorical variables, the frequencies of each category were calculated, as well as the percentage compared to the total. The level of significance was set at *p* < 0.05. For continuous variables that do not follow a normal distribution, the median and the Q1 and Q3 were calculated. A χ^2^ test was performed to find out the explanatory variables that had a significant relationship with the explained variable; these were “halftime”, “Intention”, “timepossession”, “pass”, “matchstatus” and “finalscore”. To measure the strength of this association, the size of the effect that relativized the results was calculated, and following Cohen’s criteria, it was observed that the variables “halftime”, “Intention”, “pass”, “matchstatus” and “finalscore” showed a high association level, while the variable “timepossession” showed a low association (Table 2).

A logistic regression (Table 3) was performed with these variables to find the model that would best fit the observations and that allowed obtaining a predictive value. The reference criterion was NO SUCCESS. The explained variable was “move outcome” and the parameters of the proposed model were:

Logit_(move outcome)_ = β + β_1_(halftime) + β_2_(intention) − β_3_(timepossession) + β_4_ (matchstatus) + β_5_(finalscore)+ β_6_(pass)

Hosmer and Lemeshow’s goodness-of-fit test showed a value of 0.760, indicating that the model fitted, with data that is accompanied by Nagelkerke’s R2 value of 0.456. The classification of the observations showed a sensitivity of 69.4% and a specificity of 74.3%, with a correct classification percentage of 72.6%.

The variable “halftime” (ft) increases the chance of not being successful by 1358 times. The variable “Intention” C increases the chances of non-success by 531,748 times. The variable “timepossession” decreases the chance of non-success by 0.945. The variable “finalscore” by drawing increases by 1602 times the chance of non-success and losing increases it 1740 times. The variable “pass” has an influence of 1192 on non-success.

The logistics distribution function:

$$p(\mathrm{move}\;\mathrm{outcome}\;=\;\mathrm{NO}\;\mathrm{SUCCESS}\;|X)\;=\;\;(\frac{e^{\left(-0.425+0.306(\mathrm{halftime})+6.27(\mathrm{intention})-0.56(\mathrm{timepossession})+0.176(\mathrm{pass})+0.471(\mathrm{finalscored})\right)}}{1+e^{\left(-0.425+0.306(\mathrm{halftime})+6.27(\mathrm{intention})-0.56(\mathrm{timepossession})+0.176(\mathrm{pass})+0.471(\mathrm{finalscore}\_d)\right)}})=\;0.9988211\;=\;99.80\%\;\mathrm{correct}\;\;\mathrm{clasification}\;\mathrm{of}\;\mathrm{NO}\;\mathrm{SUCCESS}.$$

Finally, an analysis was performed using the decision tree technique to predict the final result of the observed team’s attack sequence, in addition to knowing its predictive capacity with a sample of 3740 observations (Table 4, Figure 1). Two groups were formed, one training group with 60% of the observations equivalent to 2230 observations, and the other test group with 40% equivalent to 1510 observations. Cross validation was carried out using the observations of the sample group. For the construction of the model, the observations of the training group that make up the root node were used, with a loss of 316 observations and with a majority of plays ending in NO SUCCESS, which were 64.81% compared to the 35.18% who ended in SUCCESS. The next variable that the algorithm selected was “Intention”, with two routes according to the number of categories. For the “Progress” category, we worked with 2463 observations with a loss of 1150 observations, configuring an intermediate node that showed that the dominant category was SUCCESS with 53.30%, compared to a 46.69% of NO SUCCESS. The third node collects the information of the category “conserve” with 1277 observations and a loss of 3 observations. The dominant category in this case was NO SUCCESS with 99.76% and 2.34% of achieving SUCCESS. With this distribution, a first terminal node was consolidated.

The algorithm included the quantitative variable “pass”, which recoded it into two categories, one <2.5 sg. and the other ≥2.5 sg. In the first case, we worked with 266 observations with a loss of 8, where the dominant category of the variable “move outcome” was SUCCESS with a 96.99% chance of obtaining it, compared to a 3% of NO SUCCESS, configuring the second terminal node.

For the second case ≥2.5 sg the dominant criterion was NO SUCCESS, distributed in the following percentages: 48.02% for E, and 51.97% for NO SUCCESS. The next variable that the program added was ZC, differentiating between 1 (medium defensive zone) and 2 (medium offensive zone). In the case of the offensive zone, 1502 observations were analyzed with a loss of 675, with a dominant result of SUCCESS, distributed in 55.05% achieving SUCCESS and 44.94% achieving NO SUCCESS. In the case of the defensive zone, 695 observations were analyzed with a loss of 228 observations, with the result of NO SUCCESS with the following percentages: 32.80% for E and 67.19% for NO SUCCESS.

The next variable that the algorithm introduced was “timepossession”, which opened two new branches derived from ZC. The new categories were ≥20.5 sg. and <20.5 sg. that compared to the medium offensive zone showed that the first category worked with 400 observations with a loss of 143, with SUCCESS being preferred with 64.25% compared to 35.75% of NO SUCCESS, in this case a terminal node is consolidated. In the case of the category <20.5 sg, the preference is also SUCCESS with 51.72% compared to 48.27% of NO SUCCESS. In the medium defensive zone, the criteria were ≥24.5 sg. and <24.5 sg. The first case was observed 215 times with a loss of 79 observations, the dominant category was SUCCESS with 63.25%, while for NO SUCCESS it was 36.74%, constituting a terminal node. For the category <24.5 sg. with 480 observations and with a loss of 92, the dominant one was NO SUCCESS with 80.83%, and compared to a 19% SUCCESS, constituting a new terminal node.

Finally, derived from the “timepossession” node in its category <20.5 sg, the algorithm introduced the last variable “scoretime” considering two categories, w and d. In the first case, “w” 494 observations were made with a loss of 204 and the dominant criterion was SUCCESS with 58.70% and 41.29% for NO SUCCESS, consolidating a new terminal node. In the case of “d”, 608 observations were obtained with a loss of 280 and with a dominant criterion of NO SUCCESS with 53.94%, and 46.05% SUCCESS, establishing a terminal node.

The nodes that are more interesting are 2, 3, 4, 5 and 6 because they have the possibility of improving the result, while nodes 1 and 6 have a difficulty in establishing an improvement.

The following test observations were used to assess the predictive ability (Table 5):

The model predicted 941 observations as SUCCESS and these were correctly predicted as SUCCESS and 434 that in fact were incorrectly predicted as NO SUCCESS. The model also predicted 375 NO SUCCESS incorrectly as SUCCESS whereas 1990 NO SUCCESS were correctly predicted as NO SUCCESS. Therefore, the model shows a classification effectiveness of 78.36%.

## 4. Discussion

The present work was proposed with the aim of knowing how the highest-level women’s soccer teams perform regarding ball possession during games. For this, 3740 ball possessions were analyzed, made during the 52 matches held during the FIFA Women’s World Cup 2015. This competition was chosen because it is the most representative and where the highest-quality players meet. The two main novel contributions are summarized as follows: this study is one of the first works that analyzes ball possessions in high-level women’s soccer; in addition, the multivariate decision tree technique has been used, which although it had been implemented in men’s soccer in previous works [55,56,57], had not yet been tested in women’s football.

Regarding the first and second objective, it can be affirmed that 35.18% of the ball possessions end successfully (goal, shot or sending to the area), compared to the 64.8% of the possessions that were not successful. Although it is not possible to contrast these data with literature on women’s soccer, we do find that these values are consistent with the study by Maneiro et al. [24] on offensive transitions in high-performance men’s soccer.

On the other hand, it is notable that 2 out of 3 ball possessions do not succeed or end with a loss, without even reaching the rival area. In view of the data, the inefficiency is very high. Despite the fact that the choice and implementation of the playstyle is an important decision in football teams [54], and that the players and coaches dedicate many hours of training to perfect these actions, ineffectiveness rates are still very high. The literature states that some of the possible explanations for this high ineffectiveness may be due to technical aspects, poor decision-making, low concentration levels or the high speed required for these actions [58]. Nor should we forget the defensive success of the rival team in countering these actions [59,60].

Significant differences (<0.001) were also found between the possessions made between the first and second part of the match. Specifically, despite the fact that teams have more possession in the first half, the highest success rates are found in the second half. A possible explanation lies in the possible fatigue of the rival team, unable to stop possession. Another possible explanation could be found in the different playstyles adopted by the teams, as mentioned in previous works, based on match status [30,31], the area of ball possession [21] or the context of interaction of the team that makes the possession [25,29], depending on the match half.

One of the criteria that have presented a statistically significant relationship with the possession of the ball has been the “intention” criterion. Specifically, it is possible to affirm that all the ball possessions that have ended successfully (goal, shot on goal or sending the ball to the penalty area) were performed following the tactical intention of progressing towards the rival goal (<0.001). This result corroborates previous studies in men’s soccer where this criterion has been specifically collected [23] and also in women’s soccer [24], as well as in other works where it is stated that direct progression to the opponent’s goal is the most effective behavior in order to get a shot or reach the penalty area [15]. Finally, it is important to note that the success of this variable can be altered by the type of competition analyzed [61].

Regarding the time of possession in the offensive zone (opposing field) or defensive zone (own field), a statistically significant relationship (<0.001) was found between the success of possession and the duration of possession in the offensive zone. Specifically, 8 out of 10 ball possessions that ended successfully had more time in the opposite field than in their own field. These results corroborate previous works on men’s soccer [21], where it is stated that possession in the offensive zone is a predictor of success; specifically, it increases the probability of winning by 1.72 times. On the other hand, the relationship between possession of the ball and match status has also been compared in the scientific literature [16,30], concluding that the teams that are winning on the scoreboard have greater ball possession in the defensive zone. Although, in view of recent works [24], the latter seems to be inconsistent in women’s football.

The category “timepossession” also presents a statistically significant relationship in relation to ball possession. Specifically, the longest possessions are more ineffective, while those that end with a goal, a shot or a sending to the area are those that take 16 s or less. These data show the need to prioritize fast attacks over slow attacks [62].

On the other hand, the number of passes also presents a significant relationship in relation to ball possession. Specifically, the data reveals that possessions based on four pass sequences are more successful. These results corroborate recent studies in women’s soccer [25], and also works on men’s soccer where a similar number is discussed [20,24,63], although there are studies that find different results [22,64,65]. This lack of consensus is probably due to the team analyzed, as well as the type of competition, since these studies refer to league championships and league competitions.

Match status is another of criteria that presents a statistically significant relationship with ball possession (<0.016). This variable has been extensively studied in men’s soccer, with results that are not yet conclusive. In women’s soccer, match status is also a variable that modifies the behavior of ball possession [25]. The factors that affect ball possession depending on whether the team is winning, drawing or losing are multicausal, and depend on the team analyzed, the competition and the coach’s tactical intention. In this case, in view of the data, it is possible to affirm that more successful possessions are produced with a drawing scoreboard (45.6%), but an important trend is observed with the score in favor or winning (31.6%). This may be due to the playstyle, as it has been highlighted in previous works on men’s soccer [30], where the teams, when they win, stay longer in their defensive midfield, in search of a short and direct counterattack, in four passes or less.

Significant results are also found in the possessions made by the winning or losing teams at the end of the match. Specifically, winning teams had the highest percentages of successful ball possessions (52%), thus corroborating previous studies [4,13].

Finally, two types of multivariate analysis were carried out. The goal of both is to find a model that can predict the success of possessions in women’s soccer. On the one hand, the results of the logistic regression show that the model fits perfectly (0.760), although the explanatory capacity (R^2^ of Nagelkerke = 0.456) is acceptable, but moderate. One of the possible reasons is the absence of empirically collated categories associated with the possession of the ball in women’s football. Despite this, the capacity of the model is acceptable. This is the first predictive model associated with ball possessions in high-level women’s soccer.

Regarding the decision tree analysis, the criterion that presents the greatest information gain is “Intention”. Specifically, the decision to retain possession of the ball and not progress towards the rival goal is not recommended, since practically in 100% of cases it will end in no success (that is, not achieving a goal, a shot to the goal or a sending to the area). On the other hand, ball possessions that progress towards the rival goal with a sequence of two passes have a probability of reaching a goal, a shot at goal or a sending of 97% (with 266 possessions). These data confirm that teams that use fast attacks, such as counterattacks or direct attacks where a low number of passes exist, are clearly more effective than combinatorial attacks. This data corroborates previous studies in men’s soccer [24,66], and confirms the need to take advantage of the moments of role change, when going from not having the ball to having the ball, and making the quick decision to send the ball to the end zone, taking advantage of the opponent’s momentary defensive disorganization. In contrast, possessions that make three or more passes and spend more time in the offensive zone (rival midfield) show a success probability of 55%.

## 5. Conclusions

The objective of this study was to analyze how ball possessions are performed in high-performance women’s football. For this, different multivariate analyses were carried out in order to know what are the variables associated with ball possession that end with a goal, a shot or a sending to the penalty area. In addition, as a statistical novelty, the decision tree technique was implemented for the first time in women’s football.

The four main conclusions can be summarized as follows: First, two out of three possessions in women’s soccer end unsuccessfully; secondly, the criteria that presents a statistically significant relationship with the possessions that ended in success are half-time, intention, zone where the team makes possession, possession time and number of passes; thirdly, the model proposed in the logistic regression fits perfectly, with an acceptable explanatory capacity; finally, the results of the decision tree indicate that progressing to a rival goal, with very few passes, is the key to success.

## 6. Limitations

The two limitations of the present study can be summarized as follows. First, in this study only one world championship was analyzed. In order to generalize the results, it is also necessary to analyze other types of tournaments. Secondly, the paucity of scientific studies on the tactical aspects of women’s football has not made it possible to compare our results in depth with previous studies.

## Figures and Tables

**Table 1:** Dimensions that are part of the ad hoc instrument and derived category systems.

Criteria	Categories	Description
Classification phase	Groups	The “classification phase” criterion refers to the phase the team is in at the moment of being observed. This can be the group stage, round of 32, quarter-finals, semi-finals or final.
	Round of 16	
	Quarterfinals	
	Semifinals	
	Final	
Half time (Match part)	First half	This criterion refers to whether possession is made in the first or second half of the match
	Second half	
Start form	Transition	This criterion refers to whether possession begins in transition (with the ball in play), or through a set piece action.
	Set piece	
Interaction context	AR: forward versus delayed line	This criterion refers to which line recovers the ball, and against which rival line: AR (forward versus delayed line), AM (forward versus middle line), AA (forward versus forward line), MM (middle versus middle line), MR (middle versus delayed line), MA (middle versus forward line), RA (delayed versus forward line), RM (delayed versus middle line), PA (goalkeeper versus forward line).
	AM: forward versus middle line	
	AA: forward versus forward line	
	MM: middle versus middle line	
	MR: middle versus delayed line	
	MA: middle versus forward line	
	RA: delayed versus forward line	
	RM: delayed versus middle line	
	PA: goalkeeper versus forward line	
Intention	Progress	This intention can be to progress (if the observed team clearly and intentionally advances towards the opponent’s court, making 2 passes forward, or sending the ball to the opponent’s penalty area) or decides to stay (clearly and intentionally does not advance with the ball, makes passes to the end zone, or the first two passes are made backwards).
	Keep	
MD		Time the observed team keeps the ball in its defensive zone
MO		Time the observed team keeps the ball in its offensive zone
ZC		Zone in which the team maintained possession the most time
Time possession		Total possession duration
Passes		Number of total passes of the team possessing the ball
Move outcome	Goal	“Move outcome” refers to the success of ball possession: goal (goal achievement), shot (shot at goal), sending to the area (sending the ball to the opponent’s area) and no success (not success).
	Shot	
	Send to area	
	No success	
Match status	Winning	“Match status” refers to the partial results at the time of possession (this can be winning, drawing or losing).
	Drawing	
	Losing	
Final score	Win	”Final Score” refers to the final result achieved by the team that possesses the ball (win, tie or lose)
	Draw	
	Lose	

**Table 2:** Summary of the descriptives by groups of “move outcome”.

	No Success N = 2424	Success N = 1316	*p* Value	Effect Size
Phase:			0.143	
Groups	1729 (71.3%)	943 (71.7%)		
Round of 16	384 (15.8%)	176 (13.4%)		
Quarter-finals	183 (7.55%)	113 (8.59%)		
Semifinals	83 (3.42%)	51 (3.88%)		
Final	45 (1.86%)	33 (2.51%)		
Halftime:			<0.001	0.709
ft	1342 (55.4%)	624 (47.4%)		
st	1082 (72.2%)	692 (52.6%)		
Home-form:			0.894	
set-play	678 (27.8%)	363 (27.6%)		
transition	1749 (72.2%)	953 (72.4%)		
Interaction Context (COI):			.	
AA	73 (3.01%)	12 (0.91%)		
AM	31 (1.28%)	29 (2.20%)		
AR	4 (0.17%)	21 (1.60%)		
MA	274 (11.3%)	117 (8.89%)		
MM	1289 (53.2%)	847 (64.4%)		
MR	2 (0.08%)	3 (0.23%)		
PA	334 (13.8%)	111 (8.43%)		
RA	397 (16.4%)	168 (12.8%)		
RM	20 (0.83%)	8 (0.61%)		
Intention:			<0.001	0.799
Conserve	1274 (52.6%)	3 (0.23%)		
Progress	1150 (47.4%)	1313 (99.8%)		
MD	2.00 (0.00;9.00)	10.0 (5.00;17.0)	<0.001	0.255
MO	11.0 (9.00;15.0)	6.00 (2.00;11.2)	<0.001	0.410
ZC:			<0.001	0.670
1	1501 (61.9%)	249 (18.9%)		
2	923 (38.1%)	1067 (81.1%)		
Timepossession	17.0 (13.0;24.0)	16.0 (11.0;24.0)	<0.001	0.207
Pass	5.00 (3.00;6.00)	4.00 (3.00;6.00)	<0.001	0.752
Matchstatus:			0.016	0.708
dr	1163 (48.0%)	600 (45.6%)		
ls	602 (24.8%)	300 (22.8%)		
wn	659 (27.2%)	416 (31.6%)		
Finalscore:			<0.001	0.710
d	528 (21.8%)	252 (19.1%)		
l	850 (35.1%)	380 (28.9%)		
w	1046 (43.2%)	684 (52.0%)		

**Table 3:** Logistic regression model.

	B	Standard Error	Wald	gl	Sig.	Exp(B)	95% C.I. Para EXP(B)	
							Lower	Higher
halftime(1)	0.306	0.088	12.141	1	0.000	1.358	1.143	1.614
Intention(1)	6.276	0.581	116.783	1	0.000	531.748	170.355	1659.811
timepossession	−0.056	0.009	43.687	1	0.000	0.945	0.930	0.961
matchstatus			0.056	2	0.973			
matchstatus(1)	0.000	0.111	0.000	1	1.000	1.000	0.804	1.244
matchstatus(2)	0.030	0.156	0.037	1	0.847	1.030	0.759	1.399
finalscore			23.233	2	0.000			
finalscore(1)	0.471	0.119	15.805	1	0.000	1.602	1.270	2.021
finalscore(2)	0.554	0.130	18.078	1	0.000	1.740	1.348	2.246
pass	0.176	0.029	36.332	1	0.000	1.192	1.126	1.262
Constant	−0.425	0.122	12.135	1	0.000	0.54		

**Table 4:** Theoretical model tree.

(1) root 2230 316 NS (0.351871658 0.648128342)
(2) Intention = P 2463 1150 S (0.533089728 0.466910272
(4) pass < 2.5 266 8 S (0.969924812 0.030075188)
(5) pass >= 2.5 2197 1055 NS (0.480200273 0.519799727)
(10) ZC = 2 1502 675 S (0.550599201 0.449400799)
(20) time_possession >= 20.5 400 143 S (0.642500000 0.357500000)
(21) time_possession < 20.5 1102 532 S (0.517241379 0.482758621)
(42) finalscore = w 494 204 S (0.587044534 0.412955466)
(43) finalscore = d,l 608 280 NS (0.460526316 0.539473684)
(11) ZC = 1 695 228 NS (0.328057554 0.671942446)
(22) time_possession> = 24.5 215 79 S (0.632558140 0.367441860)
(23) time_possession < 24.5 480 92 NS (0.191666667 0.808333333)
(3) Intention = C 1277 3 NS (0.002349256 0.997650744)

Number of inner nodes: 6; Number of terminal nodes: 7.

**Table 5:** Theoretical model tree.

	Observation		
Prediction		S	NS
	S	941	434
	NS	375	1990

## Data Availability

The data is available to those interested, writing to the email rubenmaneirodios@gmail.com.
